# Targeting of CD34^+^CD38^-^ cells using Gemtuzumab ozogamicin (Mylotarg) in combination with tipifarnib (Zarnestra) in acute Myeloid Leukaemia

**DOI:** 10.1186/1471-2407-12-431

**Published:** 2012-09-26

**Authors:** Mays Jawad, Ning Yu, Claire Seedhouse, Karuna Tandon, Nigel H Russell, Monica Pallis

**Affiliations:** 1 Division of Haematology, University of Nottingham, Nottingham, UK; 2 Department of Clinical Haematology, Nottingham University Hospitals, Nottingham, UK

**Keywords:** Tipifarnib/Zarnestra, Gemtuzumab ozogamicin/ Mylotarg, AML CD34^+^CD38^-^ cells, DNA damage response

## Abstract

**Background:**

The CD34^+^CD38^-^ subset of AML cells is enriched for resistance to current chemotherapeutic agents and considered to contribute to disease progression and relapse in Acute Myeloid Leukaemia (AML) patients following initial treatment.

**Methods:**

Chemosensitivity in phenotypically defined subsets from 34 primary AML samples was measured by flow cytometry following 48 hr *in vitro* treatment with gemtuzumab ozogamicin (GO, Mylotarg) and the farnesyltransferase inhibitor tipifarnib/zarnestra. The DNA damage response was measured using flow cytometry, immunofluorescence and immunohistochemistry.

**Results:**

Using a previously validated *in vitro* minimal residual disease model, we now show that the combination of GO (10 ng/ml) and tipifarnib (5 μM) targets the CD34^+^CD38^-^ subset resulting in 65% median cell loss compared to 28% and 13% CD34^+^CD38^-^ cell loss in GO-treated and tipifarnib-treated cells, respectively. Using phosphokinome profiling and immunofluorescence in the TF-1a cell line, we demonstrate that the drug combination is characterised by the activation of a DNA damage response (induction of γH2A.X and thr68 phosphorylation of chk2). Higher induction of γH2AX was found in CD34^+^CD38^-^ than in CD34^+^CD38^+^ patient cells. In a model system, we show that dormancy impairs damage resolution, allowing accumulation of γH2AX foci.

**Conclusions:**

The chemosensitivity of the CD34^+^CD38^-^ subset, combined with enhanced damage indicators, suggest that this subset is primed to favour programmed cell death as opposed to repairing damage. This interaction between tipifarnib and GO suggests a potential role in the treatment of AML.

## Background

Acute myeloid leukaemia is a disease in which patients tend to respond well to remission induction chemotherapy, but relapse is common because current therapy cannot totally eradicate the leukaemic cells. Cells which survive chemotherapy may have a distinctive biology (e.g. active survival pathways, quiescent cell cycle status) compared to the bulk of cells in the clone, and/or may be protected by the bone marrow niche microenvironment in which they reside. For this reason the identification of a post-remission chemotherapy that can specifically target these cells is crucial. The CD34^+^CD38^-^ cell subset was originally thought to contain all the leukaemia initiating cells [1]. Whilst leukaemia initiating cells with a more mature phenotype have now also been found [2,3], the subset remains of particular interest since it is enriched for quiescent, chemoresistant cells which are associated with the likelihood of relapse [4-6].

Gemtuzumab ozogamicin/GO (Mylotarg) is a chemotherapeutic agent that consists of a humanised anti-CD33 antibody (Hp67.6) conjugated to N-acetyl-calicheamicin 1,2-dimethyl hydrazine dichloride, a potent enediyene antitumour antibiotic. In the MRC AML 15 trial, AML patients <60 years with favourable risk disease who were treated with GO in combination with induction chemotherapy showed a significant survival benefit, and a trend was also documented for patients with intermediate risk [7]. This benefit to good risk patients was found in a similar study conducted by the Southwest Oncology Group (SWOG) [8]. Older patients, including those with intermediate risk cytogenetics, also benefit from the addition of GO to remission induction chemotherapy [9,10]. Using an *in vitro* short-term culture system consisting of a defined “niche-like” microenvironment we previously showed that GO treatment can target CD34^+^CD38^-^ cells [11]. We therefore investigated whether CD34^+^CD38^-^ cell sensitivity to GO could be enhanced by another anti-leukaemic chemotherapeutic agent for which clinical efficacy has already been established. Several agents were examined in a preliminary study, of which tipifarnib appeared to be the most promising. Tipifarnib is an orally bio-available, nonpeptidomimetic, methylquinolinone farnesyltransferase inhibitor, exhibiting clinical activity against a number of haematological malignancies [12-14] and has shown enhanced toxicity when combined with other chemotherapeutic agents [15-17]. A Phase II trial combining tipifarnib with etoposide showed elevated complete remission (CR) rates in AML patients [18]. Tipifarnib has also been assessed in combination with idarubicin/cytarabine in a Phase I/II study and found to cause better CR duration and higher CR rates in AML patients with chromosome 5/7 abnormalities [19].

In this report we establish the efficacy of combining tipifarnib with GO *in vitro*, particularly in CD34^+^CD38^-^ AML cells, and investigate the mechanisms involved.

## Methods

### Cell samples

Blood or bone marrow samples were obtained with written informed consent from AML patients and healthy stem cell donors. Use of these samples was approved by the Nottingham 1 Ethics Committee and the Nottingham University Hospitals NHS Trust. Mononuclear cells were isolated using a standard density gradient centrifugation method with Histopaque.

### Materials

Recombinant cytokines were obtained from R&D Systems (Abingdon, UK). Phenotyping antibodies were from Becton Dickinson (Cowley, UK). GO was kindly provided by Wyeth Pharmaceuticals (PA, USA) and tipifarnib by Johnson and Johnson Pharmaceutical Research and Development (NJ, US). Unless otherwise stated, all other reagents were purchased from Sigma (Poole, UK). Stock solutions were prepared as follows: GO (1 mg/mL), daunorubicin (1 mM), vinblastine (4.5 mg/ml) and verapamil (50 mg/ml) were reconstituted in water; tipifarnib in 1 V HCl: 2 V Methanol (25 mM), cyclosporin A in 100% ethanol (25 mg/mL).

### Cell culture

#### Cell lines

The KG-1a and U937 cell lines were purchased from the European Collection of Animal Cell Cultures and the TF-1a cell line from the American Tissue Culture Collection (ATCC). U937 and TF-1a cells were maintained in RPMI 1640 with 10% foetal calf serum (FCS; First Link), 2 mmol/L L-glutamine, 100 U/mL penicillin, and 10 μg/mL streptomycin (R10). KG-1a were maintained as above but with 20% FCS. All cultures were kept at 37°C in 5% CO_2_ and all experiments were done with cell lines in log phase. Continued testing to authenticate these cell lines was done using a panel of monoclonal antibodies toward the final passage of each batch thawed.

#### Primary AML cells

Fresh or cryopreserved AML cells were cultured at 10^6^/ml for 48 hours, in triplicate in serum-free medium consisting of Iscove’s modified Dulbecco medium supplemented with 200 μg/ml transferrin, 10 μg/ml insulin, 1% L-glutamine, 2% bovine serum albumin and 10^-4^ M mercaptoethanol. Fibronectin-coated wells and serum free medium (SFM) were used as previously described [20]. For maintenance of CD34^+^CD38^-^ cell phenotype in 48 hours culture we treated AML samples in serum-free medium with immobilized fibronectin along with a combination of cytokines consisting of IL-3 (20 ng/ml), SDF-1 (100 ng/ml), SCF (50 ng/ml) and TPO (50 ng/ml).

### Phosphorylated protein detection

For the detection of the relative phosphorylation levels of 46 intracellular kinases we used the Human Phospho-Kinase Antibody Array (Catalogue # ARY003, R&D Systems, Abingdon, UK) according to manufacturers’ instructions. Proteins were visualized using chemiluminescence (Hyperfilm ECL; Amersham), scanned using a Syngene densitometer, and analyzed using the GeneSnap software (Syngene).

### Flow cytometry

#### Chemosensitivity assays and immunophenotyping

Phenotyping was carried out using antibodies to CD34, CD38, CD123 and CD33. A preliminary comparison of test/control relative fluorescence intensity (RFI) values in leukaemic samples versus those of CD34^+^CD38^-^ cells from healthy donor samples was carried out to establish cut-off points to verify the leukaemic nature of samples assessed (for CD33, n = 11, mean RFI + 2 standard deviation cut-off point = 8.67; for CD123, n = 12, mean RFI + 2 standard deviation cut-off point = 17). Flow cytometric enumeration of CD34^+^CD38^-^ and bulk AML cells were measured in leukaemic samples as previously described [20]. Briefly, two flow cytometric analyses were used in parallel for the analysis of CD34^+^CD38^-^ cell survival. One assay allowed reproducible measurement of the concentration of viable cells at the end of the experiment using 10 μg/ml 7-amino-actinomycin D (7-AAD) and fixed CD45-stained normal mononuclear cells as internal standard [21]. The second assay allowed the determination of the percentage of CD123^+^CD34^+^CD38^-^ cells within the viable population using CD34 FITC and CD38 APC and CD123 PE (or isotype controls) with 7-AAD. From these 2 analyses we calculated the concentration of leukaemic CD34^+^CD38^-^ cells from the cell count and the proportion of viable cells which are CD34^+^CD38^-^CD123^+^.

### Determination of RNA status

The method of Toba 1995 was used, using 7-AAD to label DNA and pyronin Y to label RNA [22]. RNA was also measured on unselected cells by spectrophotometry (Nanodrop 2000, ThermoScientific, UK distributor Fisher Scientific, Loughborough, UK).

### Measurement of P-glycoprotein protein and function

P-glycoprotein (Pgp) protein and function was measured using flow cytometric methodology as previously described [23]. Each assay involved labelling with the fluorescent probe or antibody of interest and with an antibody against CD45 to allow leukemic (CD45 low/side scatter low) cells to be gated. The peridinin chlorophyll protein conjugate to CD45 was chosen to avoid spectral overlap with FITC labels. Briefly, Pgp substrate efflux modulation by tipifarnib, Cyclosporin A, vinblastine and verapamil was determined in a modulation assay using rhodamine 123 based on the report by Broxterman et al. [24]. For protein measurement, MRK16 anti-Pgp (Kamiya Biomedical) antibody (30 minutes, room temperature) was used followed by 20% normal rabbit serum to block (30 minutes, 4°C) and FITC-conjugated goat anti-mouse secondary antibody (30 minutes, 4°C; Dako). U937 (Pgp negative) and KG1a (Pgp positive) were used as controls.

#### Measurement of γH2AX and phospho-Chk2(Thr68)

Cells were fixed and permeabilised using the Leucoperm kit (AbdSerotec) with an additional 10 minute incubation in ice-cold methanol after the first fixation step. For γH2A.X, 2 μg/mL mouse monoclonal antibody (Upstate) or mouse isotype control (DAKO) was added (2 hours, RT) and goat anti-mouse IgG FITC F(ab')2 secondary antibody (DAKO) was used as second layer. For phosphorylated Chk2 (Thr68), a rabbit polyclonal antibody (1:25 dilution Cell Signalling Technology) was added or not for 1 hour at RT and goat anti-rabbit FITC was used as second layer. Data were analyzed on a FACS CANTO using the DIVA software with the phosphorylation status expressed as a ratio between test and negative control antibodies (relative florescence ratio; RFI). For primary cell work, cryopreserved cells were used (65-99% blasts). These cells were counterstained with CD34 PerCP and CD38 APC. The PE channel was not used in order to maximise accuracy of FITC specific fluorescence without recourse to fine-tuning compensation for individual samples. Isotype control antibodies were used to set gates for CD34 and CD38 fluorescence.

#### Immunohistochemistry and immunofluorescence

For immunohistochemistry, cells were fixed on glass slides, labeled with γH2A.X and counted using the H score, a semi-quantitative measurement of damage foci per 100 cells, as previously described [25]. For immunofluorescence, cells were labeled as for flow cytometry and were mounted on glass slides in DAPI-containing mounting medium.

### Cytogenetics, FLT3 and NPM1 status

FLT3/ITDs were analysed by previously described methods [26]. NPM1 mutation status was identified as described by Noguera et al. [27]. Stratification into favourable, intermediate, and adverse cytogenetics groups was made according to guidelines established in Medical Research Council studies [28].

### Data output and statistical analysis

Statistical analysis was carried out using the Statistical Package for Social Sciences, version 16 (SPSS). Tests used were based on the assumption that cell line data was parametrically distributed and patient cell data non-parametrically distributed. P values of ≤0.05 were considered to represent significance. The supra-additive nature of the combination of tipifarnib and GO was made by comparing the toxicity of the combination with the sum of the toxicities of the two agents individually by Wilcoxon signed rank tests.

## Results

### Assessment of the leukaemic nature of CD34^+^CD38^-^ cells

The frequency of CD34^+^CD38^-^ cells in normal bone marrow has been calculated as 0.02 ± 0.01% mononuclear cells [29]. In 27 of our original cohort of 38 samples, the proportion of CD34^+^CD38^-^ cells was >2%, i.e. at least 100X the normal value and therefore judged to be overwhelmingly leukaemic. Of the remaining 11 samples, 7 over-expressed both CD123 and CD33 on their CD34^+^CD38^-^ cells, 1 over-expressed CD123 but not CD33 and 1 over-expressed CD33 but not CD123. In 2 samples a defining leukaemic phenotype was not found and these samples were excluded from further analysis. In addition, 2 samples had insufficient viable CD34^+^CD38^-^ cells for analysis after 48 hour culture, such that the final cohort analysed comprised 34 samples.

### Chemosensitivity to tipifarnib/GO combination in primary AML cells

**Figure 1:**
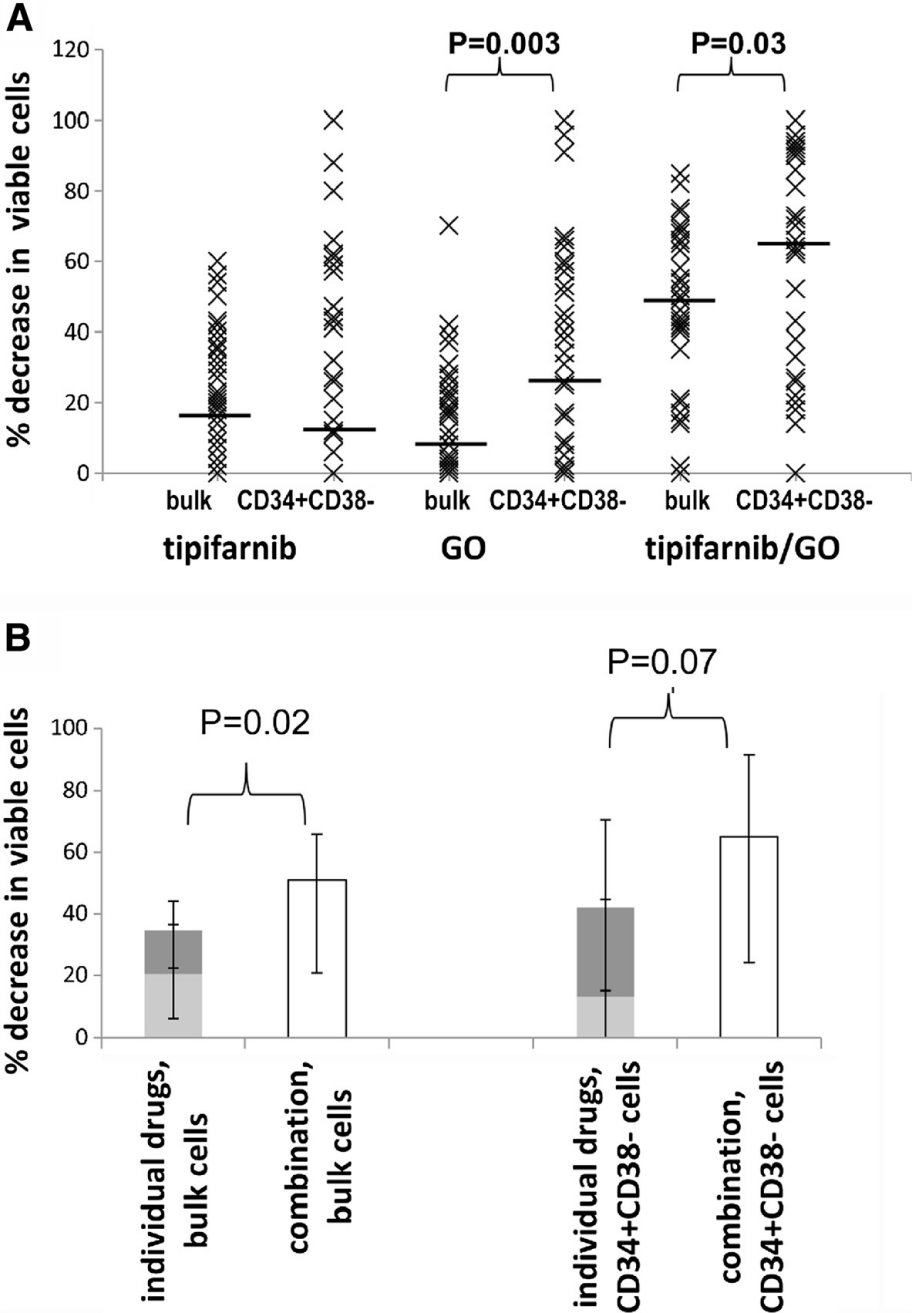
**Chemosensitivity to Tipifarnib/GO.** Percentage decrease in viable bulk and CD34^+^CD38^-^ patient cells compared to untreated controls after 48 hour drug treatment with 10 ng/ml GO, 5 μM tipifarnib or the combination. N = 34 **A**. Individual values - median values are indicated by black bars. **B**. Median values showing that overall, the drug combination is significantly more toxic (bulk cells) or as toxic (LSPC) as the sum of the individual drugs. Key: tipifarnib = light grey bars, GO = dark grey bars, combination = white bars, errors bars = interquartile range

We used an *in vitro* model comprising immobilised fibronectin, serum-free medium and a mixture of the cytokines IL-3, SCF, TPO and SDF-1 to support the survival of CD34^+^CD38^-^ cells in culture without loss of phenotype [11]. The response of primary AML blasts to GO at 10 ng/ml has previously been reported [11], and this was maintained in the current study. With insufficient CD34^+^CD38^-^ cells in most samples to study more than one concentration of each drug, we carried out a preliminary study to establish a concentration of tipifarnib (5 μM) that would induce a low level (10-30%) cell kill as a single agent (data not shown). When the cohort was expanded to 34 patient samples, tipifarnib (5 μM) treatment (48 hours) was found to induce a median 20% bulk cell kill versus 13% in CD34^+^CD38^-^ cells. As previously found [11] GO (10 ng/ml) treatment (48 hours) alone caused a greater decrease in viable cells in the CD34^+^CD38^-^ subset than in bulk cells (14% in bulk cells versus 28% in CD34 + CD38- cells, P = 0.003). The combination of 5 μM tipifarnib and 10 ng/ml GO resulted in a median bulk cell kill of 51% and median CD34^+^CD38^-^ cell kill of 65% (Tables 1 and 2 and Figure 1A). Excluding the 1 bulk cell sample and 5 CD34^+^CD38^-^ samples in which the sum of the individual toxicities of tipifarnib and GO was ≥100%, we determined that the combination was supra-additive in bulk cells (n = 33, P = 0.009). there was a non-significant trend towards a supra-additive effect in CD34^+^CD38^-^ cells (n = 29, P = 0.066, Figure 1B). Cytogenetics were available for 23 samples (Tables 1 and 2). By MRC criteria [28] most samples were of intermediate prognostic risk (n = 15). Only five samples belonged to the poor risk group and three to the good risk group, rendering any subgroup analysis on these two latter groups inappropriate. Sensitivity to the drug combination correlated strongly with sensitivity to the drugs used individually (rho = 0.7, P < 0.001 for tipifarnib and rho = 0.43, P = 0.01 for GO in bulk cells, rho = 0.61, P < 0.001 for tipifarnib and rho 0.64, P < 0.001 for GO in CD34^+^CD38^-^ cells). 

**Table 1:** Characteristics of samples used in this study grouped by responses of cells to Tipifarnib and GO: bulk cells

**AML#**	**Cytogenetics**	**FLT3 Status**	**NPM1 Status**	**CD33 MFI**	**Pgp status**	**Tip%cell loss**^**1**^	**GO%cell loss**^**1**^	**Tip/GO%cell loss**^**1**^	**Δtox**^**2**^
**#19**	normal	WT	WT	1.7	NEG	33	70	85	-
**#1**	normal	ITD	MUT	74	NEG	60	23	82	−1
**#10**	plus 8	ITD	WT	3.7	NEG	54	3	75	18
**#17**	inv(16)	ITD	WT	22	NEG	35	27	75	13
**#13**	normal	WT	WT		NEG	42	39	74	−7
**#3**	inv(16)	WT	WT	19	POS	36	0	70	34
**#15**	—	ITD	WT	3.3	POS	50	17	69	2
**#27**	complex	ITD	WT	38	NEG	0	0	68	68
**#33**	del(13)	ITD	—	198	NEG	38	19	65	8
**#14**	normal	ITD	MUT	8.7	NEG	43	4	65	18
**#32**	—	ITD	MUT	46	NEG	19	18	65	28
**#8**	normal	WT	MUT	83	NEG	20	12	63	31
**#12**	inv(16)	WT	WT	1.5	NEG	56	28	63	−21
**#26**	—	ITD	MUT		NEG	22	31	58	5
**#11**	—	ITD	MUT	43	NEG	11	1	55	43
**#30**	—	WT	WT	38	NEG	29	18	54	7
**#34**	normal	ITD	MUT	1.3	NEG	4	42	52	6
**#20**	—	WT	WT	30	POS	27	23	50	0
**#21**	del(5q)	ITD	MUT	59	NEG	40	25	49	−16
**#5**	normal	ITD	MUT	75	NEG	0	16	46	30
**#25**	dup1, add12, add16	WT	—	36	POS	18	10	43	15
**#9**	normal	ITD	MUT	8.4	NEG	19	19	42	4
**#6**	normal	ITD	MUT	0.19	NEG	31	37	41	−27
**#16**	—	—	—	51	—	21	21	40	−2
**#4**	—	ITD	MUT	89	NEG	33	0	35	2
**#35**	complex	WT	WT	18.2	POS	14	5	21	2
**#31**	normal	ITD	WT	11	POS	7	0	20	13
**#2**	—	WT	WT	3.4	POS	2	0	17	15
**#7**	plus 11	WT	WT	23	NEG	0	3	15	12
**#29**	normal	WT	MUT	140	NEG	16	0	14	−2
**#28**	complex	—	—	4.9	POS	0	2	2	0
**#18**	−5	WT	WT	2.1	POS	0	8	0	−8
**#36**	—	WT	WT	6.3	—	9	2	0	−11
**#23**	—	WT	WT	36	—	0	1	0	−1

34 AML samples used in this study ordered by magnitude of response to tipifarnib + GO (column second from the right).

^1^% of cells lost after 48 hours treatment (compared to untreated control).

^2^ Δtox = cell loss with drug combination – (cell loss with tipifarnib + cell loss with GO). No values are displayed when cell loss with tipifarnib + cell loss with GO= > 100%.

**Table 2:** **Characteristics of samples used in this study grouped by responses of cells to Tipifarnib and GO: CD34**^+^**CD38**^-^**cells**

**AML#**	**%CD34**^+^**CD38**^-^	**CD123 MFI**	**CD33 MFI**	**Tip**% **cell loss**^**1**^	**GO%cell loss**^**1**^	**Tip/GO%cell loss**^**1**^	**Δtox**^**2**^
**#13**	3	92	—	88	96	100	-
**#19**	3.4	20	1.4	66	91	100	-
**#1**	40	51	193	61	40	96	-
**#10**	7.6	12	0.5	80	26	94	-
**#33**	0.3	46	143	26	67	94	1
**#4**	1.7	35	437	57	57	93	-
**#14**	45	319	9.7	59	25	93	9
**#32**	33	62	51	0	45	92	47
**#11**	2.5	35	61	12	53	91	26
**#9**	0.3	34	13	32	57	90	1
**#34**	2.6	70	1.5	44	31	86	11
**#16**	2.3	1.8	92	0	59	81	22
**#17**	7	4.9	16	21	51	81	9
**#8**	17	53	72	0	0	73	73
**#15**	78	23	3.4	47	16	72	9
**#5**	16	76	74	0	60	70	10
**#6**	0.2	19	57	0	66	66	0
**#20**	2.4	173	31	11	43	64	10
**#3**	9	8.4	12	15	0	63	48
**#35**	3.7	9.2	7.2	62	1	62	−1
**#31**	0.8	55	9.6	41	38	52	−27
**#26**	2.4	17	9	43	0	43	0
**#25**	12	42	3.2	15	2	38	21
**#18**	85	20	1.9	0	8	33	25
**#29**	0.9	62	31	0	64	27	−37
**#27**	14	20	100	0	0	26	26
**#2**	54	20	3.1	6	5	18	7
**#36**	53	6	6.2	27	9	14	−22
**#7**	23	11	15	0	5	0	−5
**#12**	0.3	29	7.5	0	34	0	−34
**#21**	6.2	24	53	0	0	0	0
**#23**	0.2	4.2	20	0	2	0	−2
**#28**	6	17	3.2	0	0	0	0
**#30**	2.4	90	34	0	0	0	0

34 AML samples used in this study ordered by magnitude of response to tipifarnib + GO (column second from the right).

^1^% of cells lost after 48 hours treatment (compared to untreated control).

^2^ Δtox = cell loss with drug combination – (cell loss with tipifarnib + cell loss with GO). No values are displayed when cell loss with tipifarnib + cell loss with GO= > 100%.

### The tipifarnib/GO combination induces a DNA damage response pathway

**Figure 2:**
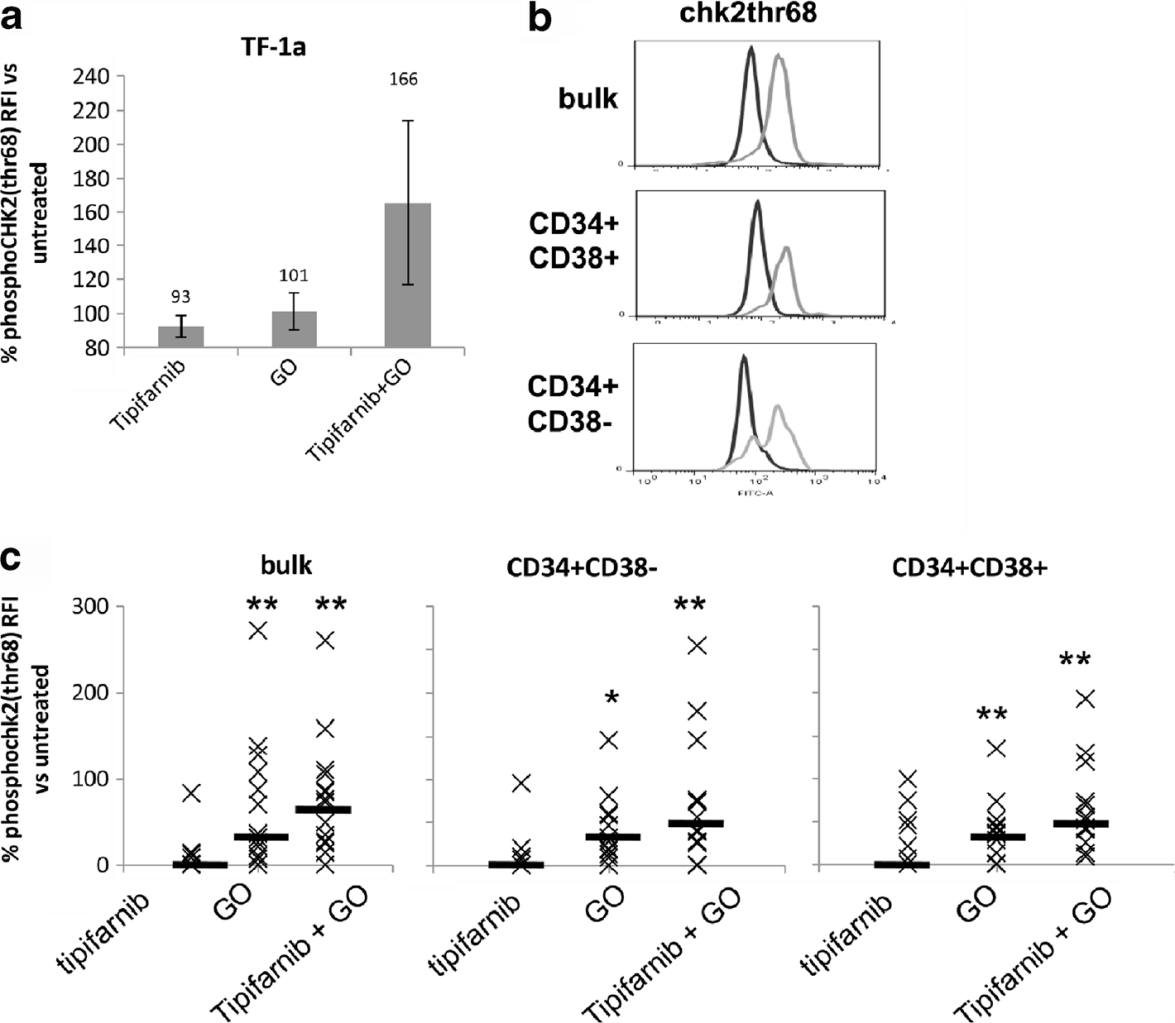
**Phospho-chk2(thr68) in treated cells.** Cells were treated with 10 ng/ml GO, 5 μM tipifarnib or the combination. Data are expressed as phospho-chk2 test – isotype control fluorescence intensity compared to untreated cells. (**a**) TF1A cells, n = 3, treated for 24 hours. (**b**) Flow cytometric histograms illustrating phospho-chk2 in bulk cells, CD34^+^CD38^-^ and CD34^+^CD38^+^ subsets in a patient sample: dark histogram = untreated cells, pale histogram = cells treated with tipifarnib + GO. (**c**) Summary chart of phospho-chk2 in primary AML; n = 14 bulk, n = 12 CD34^+^CD38^-^ and CD34^+^CD38^+^ subsets, treated for 48 hours. Median values are shown by a solid bar. * represents a P value compared with untreated cells of <0.05 and ** represents P < 0.01

In order to probe for factors which might support supra-additive killing of AML cells by the combination of GO and tipifarnib, a preliminary study was carried out in which the expression of 46 known human phospho-kinase proteins was measured in the CD34^+^CD38^-^ TF-1a cell line after incubation with tipifarnib, GO and the combination. Of the 46 phosphoproteins measured, only chk2 phosphorylation was noticeably increased by the GO + tipifarnib combination compared to each treatment alone (data not shown). High expression of phosphorylated chk2 with the drug combination was confirmed by flow cytometry in TF-1a cells, despite there being little or no chk2 phosphorylated by the drugs individually (Figure 2a). GO alone induced chk2 phosphorylation in primary cell culture in bulk cells and in the CD34^+^CD38^-^ and CD34^+^CD38^+^ subsets (Figure 2b,c). No chk2 activation was observed following tipifarnib treatment alone. However, the highest level of chk2 activation was seen with the drug combination in bulk cells as well as CD34^+^CD38^-^ and CD34^+^CD38^+^ subsets.

**Figure 3:**
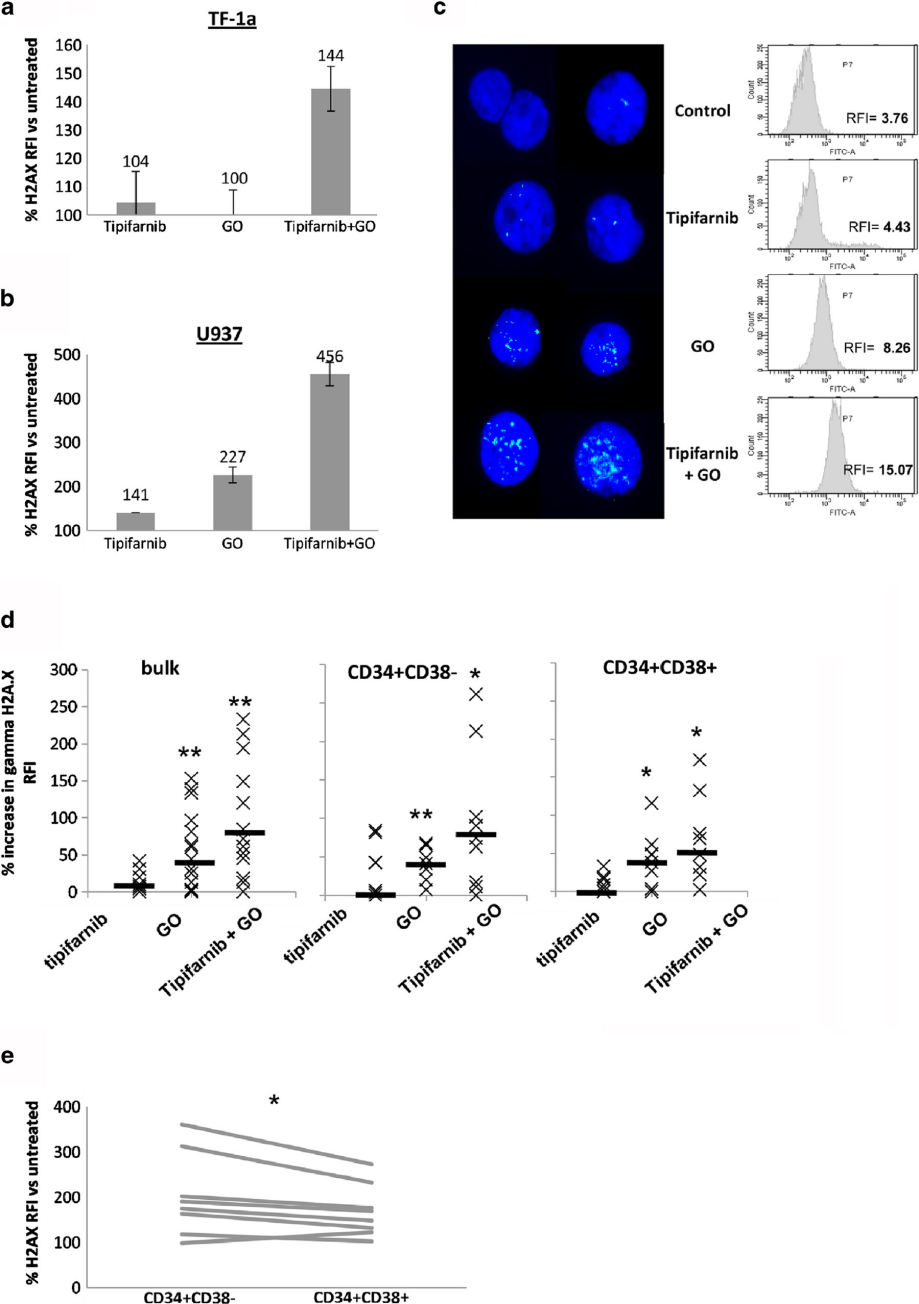
**γH2AX in treated cells.** Cells were treated with 10 ng/ml GO, 5 μM tipifarnib or the combination for the indicated times. FACs data are expressed as γH2AX test – isotype control fluorescence intensity compared to untreated cells. (**a**,**b**) Summary charts for TF-1a and U937 cells respectively cells after 48 hours’ treatment (**c**) Illustration of immunofluorescence and FACs plots showing increases in focal (immunofluorescence) and total (flow cytometry) γH2AX following 24 hours of treatment in U937 cells. (**d**) Summary charts of flow cytometric values for primary AML: bulk (n = 14); CD34^+^CD38^-^ (n = 9); CD34^+^CD38^+^ (n = 8). Median values are shown by a solid bar. * represents a P value compared with untreated cells of <0.05 and ** represents P < 0.01. (**e**) Line graph showing γH2AX induction in CD34^+^CD38^-^ cells compared to CD34^+^CD38^+^ cells in each of 8 samples

To further investigate the DNA damage response pathway, we measured the damage recognition and response protein γH2AX. In TF-1a, as seen with chk2, γH2AX was only induced by the combination, not the individual drugs (Figure 3a). U937 cells, which are sensitive to both agents, were used to illustrate that the flow cytometric method gives rise to a similar pattern of increased γH2AX as determination of foci by immunofluorescence. In these cells the combination induced considerably more γH2AX after 24 hours’ treatment than individual tipifarnib or GO treatments (Figure 3b,c). The induction of γH2AX in primary AML samples was also found to be greatest when the combination of tipifarnib + GO was used (Figure 3d). Furthermore, in primary cells, 7/8 samples studied showed higher induction of γH2AX expression occurring in the primitive CD34^+^CD38^-^ compartment compared to the more mature CD34^+^CD38^+^ cells (P = 0.035, Figure 3e).

### Impaired resolution of damage foci in dormancy-enriched leukaemia cells

**Figure 4:**
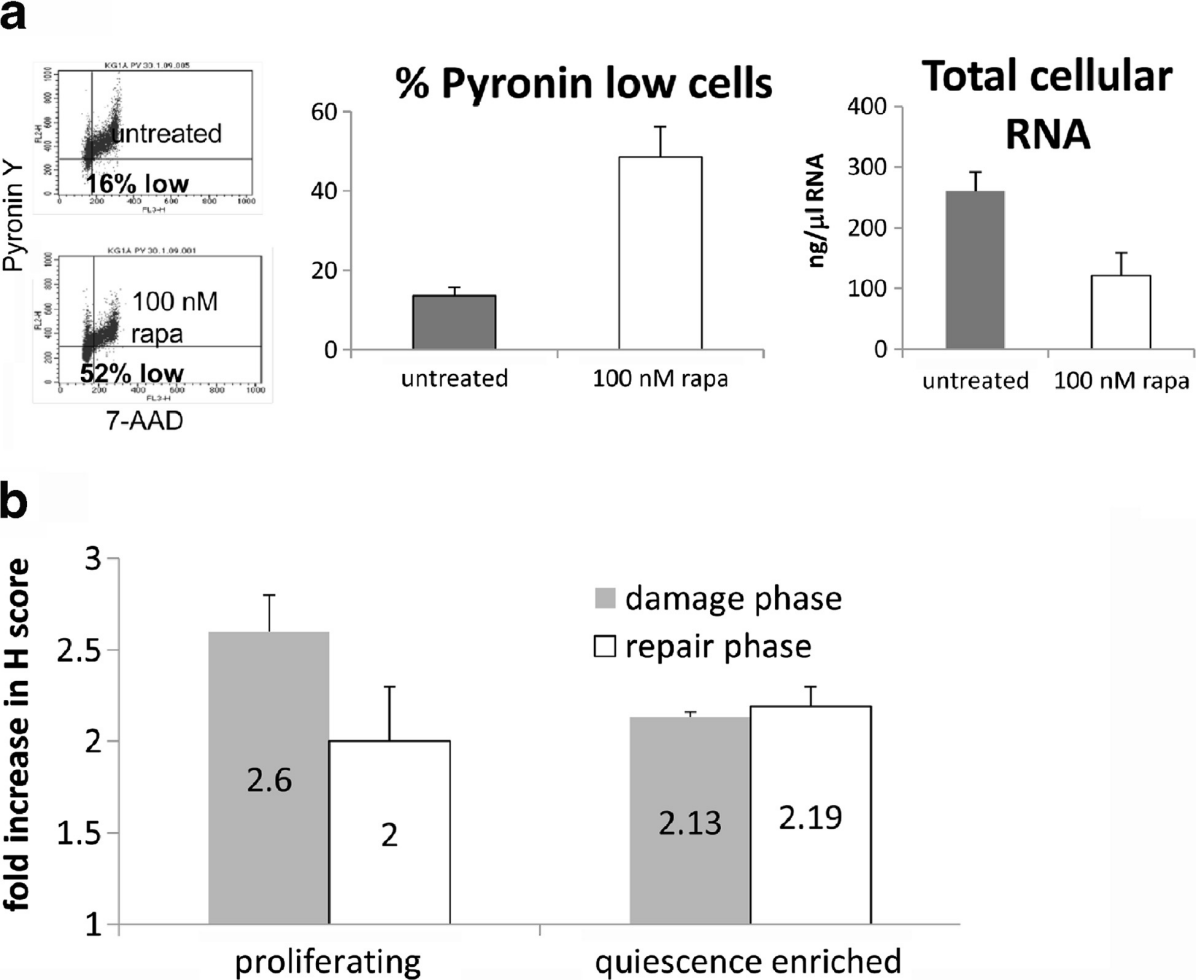
**Differential resolution of γH2A.X foci in proliferating and quiescence-enriched KG1a cells.** (**a**) Evaluation of cellular RNA content after treatment with 100nM rapamycin for 48 hours. (i) Flow cytometric dotplots indicating the percentage of cells which are low in RNA (Pyronin Y) as well as low in DNA (7-AAD); (ii) Mean and standard deviation of 5 independent assays; (iii) The total RNA content of lysed cells, measured by spectrophotometry (mean and standard deviation of 3 independent assays). (**b**) Proliferating and quiescence-enriched KG-1a cells were treated with 3 μM daunorubicin for 2 hours. Cells were then washed twice in ice cold RPMI at 4°C and were re-suspended in fresh culture medium and placed back in the incubator to allow a 2 hour repair period. γH2AX foci were measured by immunohistochemistry using the H score as described. Values correspond to the mean +/− standard deviation for the increase in γH2A.X foci compared with untreated controls, n = 3

CD34^+^CD38^-^ leukaemia cells are largely quiescent [4,30] and reported to be resistant to chemotherapeutic drugs [4,5]. However, we have shown sensitivity to tipifarnib + GO in this subset, together with enhanced γH2A.X expression. γH2A.X induction is associated with double strand breaks and initiates the homologous recombination repair pathway [31,32] which is only functional in proliferating cells. To confirm that dormant CD34^+^CD38^-^ cells are sensitive to drugs that induce a double strand break response, we compared the DNA damage response in proliferating and non-proliferating CD34^+^CD38^-^ leukaemia cells by inducing damage in CD34^+^CD38^-^ KG-1a AML cells which had been enriched for dormancy by inhibition of the mTOR pathway. In contrast to cells enriched for dormancy by serum withdrawal, the mTOR inactivation method produced cells that remained 100% viable over several days (data not shown). Low RNA content is a hallmark of quiescent leukaemic stem/progenitor cells [33], and rapamicin-treated KG-1a cells displayed a major loss of RNA, measured as 3.5fold increase in Pyronin Y^low^ cells, from 13.6 to 48.6% cells, and a decrease in average RNA per cell (measured by spectophotometry) of 54% (Figure 4a). We treated the proliferating parent and dormancy-enriched KG-1a cells with daunorubicin. The reason to use daunorubicin rather than GO in this experiment is that daunorubicin induces DNA damage rapidly [26] and provides a clear-cut model for monitoring damage induction and resolution before the onset of confounding apoptosis. So, whereas cell lines had been exposed to GO for 24 hours before analysis of γH2A.X expression, the KG-1a cells were exposed to daunorubicin for just 2 hours. Immunocytochemistry was used in order to measure the DNA damage response (DDR) after 2 hours’ treatment with daunorubicin with or without an additional 2 hours of incubation following drug withdrawal. The γH2AX antibody was used as a marker of the DDR: H-scores were recorded to demonstrate the extent of nuclear damage foci as previously reported [25]. As expected, there were more γH2A.X foci in proliferating cells compared with quiescence-enriched cells after daunorubicin treatment (Figure 4b). Strikingly, however, when the drug was removed and cells were allowed two hours to repair, the quiescence-enriched cells were completely unable to repair daunorubicin-induced damage. These data demonstrate that inhibition of proliferation can allow the accumulation of unrepaired damage and therefore indicate a vulnerability in dormant cells. 

### Investigation of additional factors that may affect relative chemosensitivity to the Tipifarnib/GO combination

#### Pgp status

**Figure 5:**
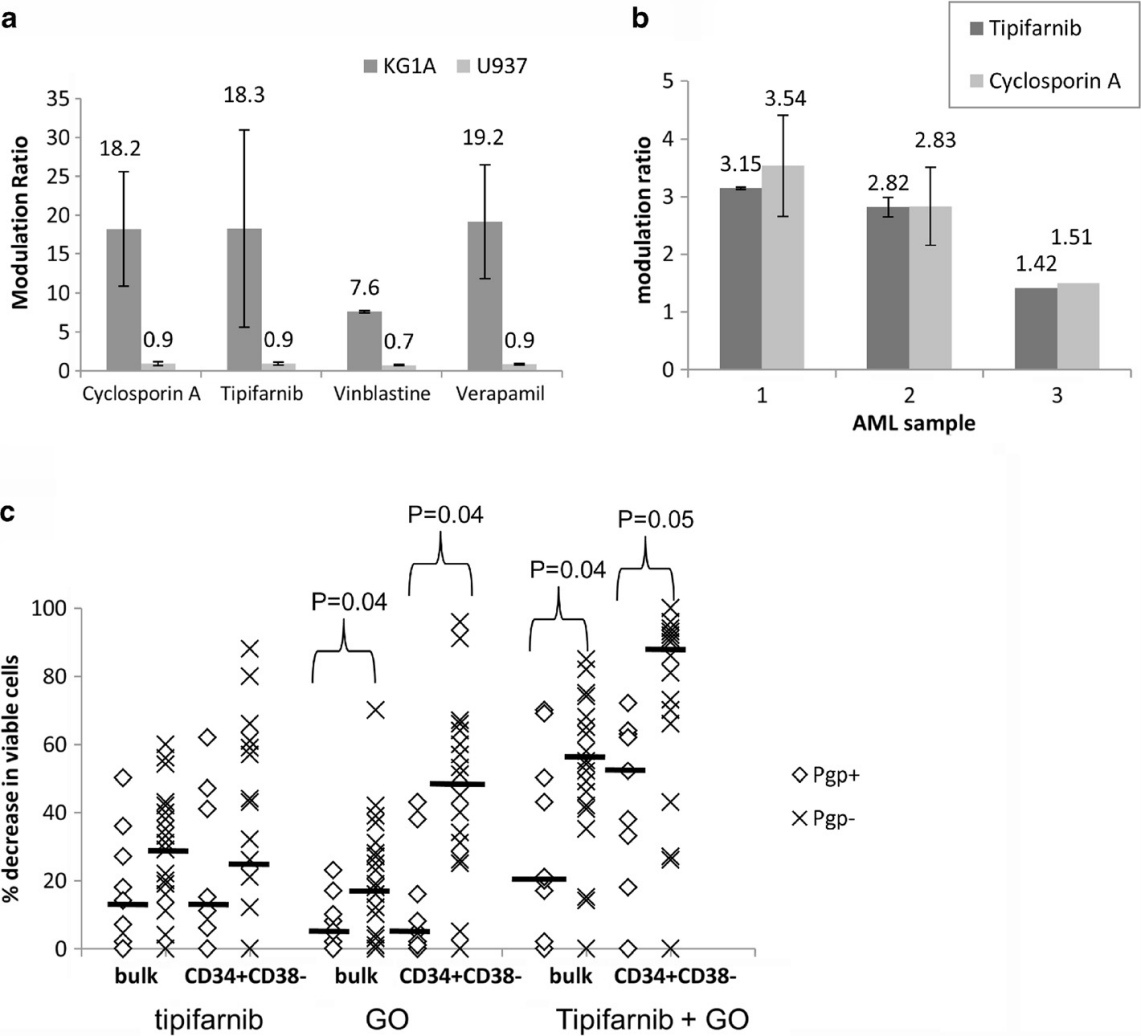
**Pgp function is inhibited by tipifarnib.** (**a**) Rhodamine 123 modulation ratio 75 minutes after treatment is shown in Pgp positive (KG-1a) and Pgp negative control (U937) cells treated with 2.5 μg/ml cyclosporin A, 5 μM vinblastine, 20 μM verapamil or 5 μM tipifarnib, mean +/− standard deviation of three separate assays (**b**) Rhodamine modulation by tipifarnib compared to cyclosporin in three different primary AML samples. (**c**) Percentage loss of viable cells is shown following 48 hour culture of nine Pgp positive and twenty three Pgp negative primary samples. Median values are shown by solid bars

Where cells were available, we measured the Pgp status of primary AML samples (31/34 samples, 9 Pgp positive and 22 Pgp negative, Tables 1 and 2). GO resistance in AML blasts is associated with Pgp over-expression [11,34]. In contrast, tipifarnib has been associated with inhibition of Pgp-mediated drug efflux [15,35]. Flow cytometry was used to evaluate the effects of tipifarnib on Pgp-mediated drug efflux using the fluorescent probe rhodamine 123 as a substrate. We compared the Pgp inhibitory activity of tipifarnib with the more commonly used Pgp inhibitors, cyclosporin A, vinblastine and verapamil. Our results indicate that Pgp inhibition by tipifarnib is well within the range of that exhibited by other Pgp inhibitors (Figure 5a), confirming Pgp-reversal activity by tipifarnib. Single tipifarnib and cyclosporin A treatments of three primary AML cells showed a very close correspondence between modulation by tipifarnib and cyclosporin A, (Figure 5b), indicating similar potency between the two agents in inhibiting Pgp activity. 

As expected from our previous study [11], Pgp positive cells were relatively insensitive to GO treatment alone compared to Pgp negative cells (P = 0.038 and P = 0.044 for bulk and CD34^+^CD38^-^ cells, respectively, Figure 5c). The drug combination also favoured Pgp negative samples (P = 0.038 for bulk cells and P = 0.053 for CD34^+^CD38^-^ cells). Our data neither supports nor contradicts the hypothesis that tipifarnib is acting in part as a Pgp inhibitor: in CD34^+^CD38^-^ cells median cell kill in the 9 Pgp + samples increased from 15% with tipifarnib and 5% with GO to 52% with the combination, but an increase was only recorded in 6/9 samples and did not reach statistical significance.

#### FLT3, NPM1, CD34^+^CD38^-^ cell burden, CD123 and CD33 expression

FLT3 status, nucleophosmin (NPM1) status and CD33 expression did not affect sensitivity to individual drugs or drug combinations (P > 0.05). Strikingly, although GO sensitivity in CD34^+^CD38^-^ cells was inversely correlated with the percentage of CD34^+^CD38^-^ in the sample (P = 0.035), this effect was absent in tipifarnib-treated cells (P = 0.82) and the tipifarnib/GO combination (P = 1).

## Discussion

Despite advances in our understanding of the mechanisms of leukaemogenesis, AML still remains a disease with poor outcome, especially because of disease relapse. This is due to chemoresistant cells surviving the initial exposure to cancer chemotherapy. The characterisation of agents that specifically target relapse-causing cells within their protective niche microenvironment is essential to achieve complete eradication of minimal residual disease cells in AML. We have previously reported that GO targets CD34^+^CD38^-^ AML subpopulation enriched for stem and progenitor cells [11]. Moreover the recent finding that the addition of GO to standard induction chemotherapy significantly increases disease free survival and reduces relapse risk in two major multi-centre trials [9,10] suggests an *in vivo* effect for GO in targeting cells contributing to minimal residual disease. The other drug in the combination we have studied is tipifarnib, which is clinically available for AML treatment and efficacy of which has been established [19,36]. However, no previous study has attempted to combine these two chemotherapeutic agents. Using 34 primary AML samples, we showed that the combination of GO and Tipifarnib is successful at not only targeting the bulk cells but even more so the CD34^+^CD38^-^ cell fraction under protective “niche-like” conditions (Figure 1). Whilst the CD34^+^CD38^-^ leukaemia stem and progenitor cell-enriched phenotype is not the only cell subset to initiate leukaemia in transplantation models, this subset is quiescent, chemoresistant and its presence predicts for poor outcome in AML [4-6].

We have demonstrated a DNA damage response to GO alone and to the tipifarnib + GO combination. The DNA damage response indicator γH2A.X (Figure 3) and chk2-phosphothreonine68 (Figure 2) were elevated in CD34^+^CD38^-^ cells as well as in bulk cells. Leukaemic CD34^+^CD38^-^ cells tend to be dormant [4,30], and despite its canonical role as a checkpoint kinase, chk2 is known to respond to damage in dormant cells [37]. It must be borne in mind that the damage response can favour either repair or apoptosis. Thus, whereas CD34^+^CD38^-^Lin-cord blood cells have a delayed double strand break response compared to CD34^+^CD38^+^ progenitors [38], chk2 knockdown was found to impair, rather than enhance, apoptosis in stem cells [39]. This is of particular interest because Chk2 inhibitors have been developed for the express purpose of sensitising cancer cells to chemotherapy drugs, but in contrast to chk1 inhibitors, these do not have proven efficacy, and in some situations have been found to inhibit rather than enhance apoptotic pathways [40]. The data from Dick and colleagues suggest that apoptosis is favoured by (largely dormant) haemopoietic stem cells with activated chk2. Our data suggest that the same may be true of leukaemic cells, and moreover, by including GO in a combination which induces DNA damage, the CD33^+^CD34^+^CD38^-^ cells over-expressed in leukaemic [41,42], but not in normal, adult bone marrow can be targeted.

To specifically examine whether the DNA damage response is enhanced or impaired in dormant CD34^+^CD38^-^ cells, we studied mTOR-inhibited KG-1a cells treated with daunorubicin, and found that these incur a smaller double strand break response than proliferating cells during a short pulse of drug, but are almost totally unable to repair the damage, such that, by two hours post-treatment, they have a higher burden of γH2A.X foci than proliferating cells. Hence, our data confirm that a DNA damage response can be induced in dormant CD34^+^CD38^-^ leukaemia cells. However, in the case of primary cells treated *in vitro* with GO and tipifarnib, another potential scenario is predicated on the fact that leukaemic CD34^+^CD38^-^ cells, driven by autocrine and paracrine cytokines [33], frequently re-enter the cell cycle. Thus we cannot conclude definitively that the observed damage responses are occurring in truly quiescent cells.

GO alone induced high chk2 phosphorylation in primary cell culture in bulk cells and in the CD34^+^CD38^-^ and CD34^+^CD38^+^ subsets, consistent with a previous finding [43]. In contrast, tipifarnib did not appear to induce a double strand break response as a single agent. However tipifarnib sensitivity is associated with deficiency of the short patch single strand break repair molecule aprataxin [18,44] and tipifarnib has been reported to induce DNA damage via reactive oxygen species [45]. Of interest here is that both calicheamicin and reactive oxygen species produce 3’phosphoglyolate (3’PG) blocking groups in DNA, which, if not processed efficiently, will result in strand breaks [46]. The combination of a 3’PG-bistrand DNA damage inducer and a reactive oxygen species inducer may result in complex locally damaged sites which in turn may contribute to the large increase in the double strand break response seen with the drug combination. Tipifarnib has many potential molecular targets in AML cells [47] and we acknowledge that any one of these may contribute to the mechanism of its activity and interaction with GO. However, the importance of the DDR in the interaction emerged strongly from our initial phosphokinome profiling.

Sensitivity to GO + tipifarnib *in vitro* varied from 0% to 100% in our cohort. We reported strong correlations between the toxicity induced by the combination and the toxicities of the individual drugs. The lack of relationship between CD33 expression levels and GO toxicity was unsurprising, given that this has already been explored extensively, with evidence for [48] and against [49,50] the expected association. Jedema and colleagues have previously noted that the failure of excess free CD33 antibody to block GO-mediated toxicity in primary AML blasts is concentration-dependent, and occurs at concentrations greater than 1 ng/ml (we used 10 ng/ml in the current study) [50]. These authors found evidence for antibody uptake by endocytosis, and their work was predicated on the finding in a clinical study that CD33 expression did not clearly correlate with GO response. Walter and colleagues found that CD33 expression had a statistically significant correlation with outcome in 276 AML patients treated with GO monotherapy, but this effect was small and therefore had minimal predictive value [48].

We and others have previously reported a role for Pgp in GO sensitivity [11,34]. In the current study we have shown that a significant association remains when tipifarnib is used together with GO, despite the role of tipifarnib as a Pgp inhibitor. Normal haematopoietic CD34^+^CD38^-^ cells over-express Pgp, but we have shown previously that leukaemic CD34^+^CD38^-^ subsets express Pgp at the same levels as more mature cells in the sample [11], so we could not expect Pgp over-expression to account for differences in chemosensitivity between bulk cells and CD34^+^CD38^-^ cells in the same individuals.

## Conclusions

In summary, this study is the first to assess combining GO with tipifarnib, both of which separately have shown clinical efficacy in AML. This drug combination targets AML cells *in vitro* including the CD34^+^CD38^-^ cells associated with chemoresistance. The activation of a DDR pathway by GO is amplified by its combination with tipifarnib. Based on our *in vitro* data we suggest assessing this two drug combination in the clinic as a potential chemotherapy regimen in the treatment of AML.

## Competing interest

The authors indicate no potential competing interest.

## Authors’ contributions

MJ and CS designed and performed research, analyzed data and wrote the paper. MP designed and facilitated the research, analysed data and wrote the paper. NR designed and facilitated the research and edited the paper. KT and NY performed research and analysed data. All authors read and approved the final manuscript.

## Pre-publication history

The pre-publication history for this paper can be accessed here:

http://www.biomedcentral.com/1471-2407/12/431/prepub

## Author details

^1^Division of Haematology, University of Nottingham, Nottingham, UK.^2^Department of Clinical Haematology, Nottingham University Hospitals, Nottingham, UK.
